# Integration of Environmental Friendly Perovskites for High-efficiency White Light-emitting Diodes

**DOI:** 10.1186/s11671-019-2980-4

**Published:** 2019-05-02

**Authors:** Hanxin Liu, Chun Sun, Zhiyuan Gao, Chong Geng, Shuangshuang Shi, Le Wang, Sijing Su, Wengang Bi

**Affiliations:** Tianjin Key Laboratory of Electronic Materials and Devices, School of Electronics and Information Engineering, Hebei University of Technology, 5340 Xiping Road, Tianjin, 300401 People’s Republic of China

**Keywords:** Quantum dots, perovskite, WLED, doping

## Abstract

Perovskite quantum dots (QDs) have been widely used in white light-emitting diodes (WLEDs), due to their high quantum yield (QY), tunable bandgap, and simple preparation. However, the red-emitting perovskite QDs are usually containing iodine (I), which is not stable under continuous light irradiation. Herein, perovskite-based WLED is fabricated by lead-free bismuth (Bi)-doped inorganic perovskites Cs_2_SnCl_6_ and less-lead Mn-doped CsPbCl_3_ QDs, which emits white light with color coordinates of (0.334, 0.297). The Bi-doped Cs_2_SnCl_6_ and Mn-doped CsPbCl_3_ QDs both show excellent stability when kept in the ambient air. As benefits from this desired characteristic, the as-prepared WLED shows excellent stability along with operating time. These results can promote the application of inorganic perovskite QDs in the field of WLEDs.

## Introduction

Among solid-state lighting technology, white light-emitting diodes (WLEDs) are excellent candidates to replace incandescent lamps for their merits of high-energy conservation, long lifetime, high luminous efficiency, and polarized emissions [1]. In general, WLEDs were recognized as one kind of the economical and efficient solid-state lighting sources [2, 3]. QD-LED technology is gradually developed over the past few years, because of high stability and high quantum yield (QY) of quantum dots (QDs) [4]. Recently, perovskites have attracted much attention, and they have been applied in many different fields [5–15]. Perovskite solar cells with power conversion efficiency (PCE) exceeding 23% have been achieved because of their excellent absorption (Abs) coefficients, long carrier diffusion lengths, and high carrier mobilities [5–7]. CsPbBr_3_-encapsulated PbSe wires have shown good optoelectronic performance including high responsivity (~ 10^4^ A W^−1^) and fair response speed (~ ms), demonstrating their great potential application in photodetection fields [8–10]. Additionally, perovskites have been introduced into photonic devices. The remarkable features of perovskite, such as rich phase compositions and low-temperature solution process ability, made perovskite can be applied in transistors [5]. Low-threshold amplified spontaneous emission and lasing can be realized by CsPbX_3_ QDs [11]. Most of all, perovskite QDs are the most promising material among QDs for LED application, due to high QY (up to > 90%), intense photoluminescence (PL), simple preparation procedure, and highly tunable bandgaps (from 1.46 to 2.50 ev) [11–16]. However, the anion-exchange reaction between perovskites and instability of iodine (I)-containing perovskite largely restricted the development of perovskite QDs toward WLED application. Sun et al. proposed to use silica encapsulation to enhance stability and avoid anion exchange [17]. The air stability of perovskite QDs was enhanced greatly, but the WLED stability is not good enough because of the significant dropping of red light. Then, Shen et al. used the anthracene shell to protect red-emitting perovskite QDs, which enhanced the LED stability against current [18]. Zhong and coworkers directly used red-emitting K_2_SiF_6_:Mn^4+^ phosphor to replace iodine (I)-containing perovskite QDs [19]. Sun and coworkers also presented the same method to enhance the stability of LED [20]. Due to the sharp emission lines of perovskite QDs, they usually are used in backlight display application with blue-emitting LED chips [21, 22]. These WLEDs are not proper for solid-state lighting, because their CRI is quite low. Recently, several reports have prepared single-phase phosphor of perovskites, which possess broad linewidths. However, the QYs of this kind of material are relatively low [23–25]. Another great problem is that the perovskite QDs contain lead, which is poison to health and environment [26, 27]. With the growing concerns about this risk, restrictions have been made to limit the use of Pb in consumer electronics. A lot of efforts have been done to explore and replace lead with less toxic elements like Sn, Ge, Bi, and Sb, which have analogous electronic band structure [28–30]. However, their optoelectronic properties cannot be comparable with the Pb-based counterparts. Doping less toxic elements into perovskite lattice has been an alternative route, which can introduce new optical, electronic, and magnetic properties [31, 32]. For example, Zhang et al. have prepared Mn-doped perovskite QDs with QYs up to 54% and the highest Mn substitution ratio was 46% [31]. Tang and coworkers reported Bi-doped lead-free inorganic perovskites. After doping Bi, the PLQY of Bi-doped Cs_2_SnCl_6_ is enhanced to 78.9% [33].

In this work, we introduce Mn-doped CsPbCl_3_ QDs and Bi-doped Cs_2_SnCl_6_ as the orange emission light and blue emission light to fabricate high-performance WLEDs. These two materials both can be excited by UV light and exhibit high QYs under UV light. They also contain the same anion Cl, which avoids the anion exchange reaction during the mixing process. Besides, it is worth to note that the emission linewidths of these two perovskites are very broad, which facilitate to form a continuous spectrum. In a WLED with a CCT of 5311K, the color coordinates of (0.334, 0.297) and CRI of 80 were achieved. Most of all, this WLED showed excellent stability against increasing currents and working time.

## Methods

### Materials and Chemicals

Cesium carbonate (Cs_2_CO_3_, 99.9%), lead (II) chloride (PbCl_2_, 99.999%), cesium chloride (CsCl, 99.99%), oleic acid (OA, 90%), and 1-octadecene (ODE, 90%) were obtained from Alfa Aesar. Manganese chloride tetrahydrate (MnCl_2·_(H_2_O)_4_, 99.99%), oleylamine (OAm, 80–90%), and tin chloride (SnCl_2_, 99.99%) were purchased from Aladdin. Bismuth chloride (BiCl_3_, 99.99%) and polymethyl methacrylate (PMMA) were obtained from Macklin. Hydrochloric acid (HCl, 37 wt.% in water) was purchased from Sinopharm Chemical Reagent Co., Ltd. Methanol (99.5%) was obtained from Kermel. Toluene (99.0%) and ethyl acetate (99.5%) were purchased from Concord. Hexane was obtained from Beijing Chemical Factory.

#### Synthesis Processes

##### Preparation of Cs-oleate

The cesium-oleate solution was prepared according to the approach by Kovalenko and co-workers [31]. In brief, 0.8 g of Cs_2_CO_3_, 2.5 mL of OA, and 30 mL of ODE were loaded in a three-neck flask and dried under vacuum at 120 °C for 1 h. Next, the flask was switched to N_2_ atmosphere and heated to 150 °C until all the Cs_2_CO_3_ dissolved.

##### Synthesis of Mn-doped CsPbCl_3_

The Mn-doped CsPbCl_3_ was synthesized by hot injection method. Typically, 0.0615 g of PbCl_2_, 0.08 g of MnCl_2_ (H_2_O)_4_, 1 ml of OAm, 1 ml of OA, and 5 ml of ODE were added to a 25-mL three-neck flask and dried in a vacuum at 120 °C for 1 h. And then, the flask was heated up to 180 °C under nitrogen. At this temperature, 0.5 mL of dried OAm and 0.5 mL of dried OA were subsequently injected to solubilize the Pb and Mn sources. Then, 0.4 mL of Cs-oleate was swiftly injected, and after 5 s, the solution was cooled with an ice bath. The QDs were precipitated with hexanes and ethyl acetate by the ratio of 1:3. Then, the solution was centrifuged at 5500 rpm for 5 min. After centrifugation, the precipitates were dispersed in toluene.

##### Synthesis of Bi-doped Cs_2_SnCl_6_

The Bi-doped Cs_2_SnCl_6_ was synthesized by hydrothermal reaction method. Typically, 0.337 g of CsCl, 0.189 g of SnCl_2_, 0.032 g of BiCl_3_ powders, and 4.0 mL of 37% hydrochloric acid were sealed into a Teflon-lined autoclave (30 mL) and heated at 220 °C for 20 h. After the reaction, the autoclave was slowly cooled to room temperature, and a white crystal of Bi-doped Cs_2_SnCl_6_ could be separated by centrifugation (3000 rpm, 2 min).

##### Fabrication of LED Devices

UV-LED chips with an emission peak wavelength centered at 365 nm were purchased from Shine On Corp. In a typical preparation, a certain amount of Bi-doped Cs_2_SnCl_6_ powder was mixed with PMMA/toluene solution and coated onto the UV-LED chip. Next, Mn-Doped CsPbCl_3_ QD solution was added into a 1-ml transparent PMMA/toluene solution. After that, the Mn-doped CsPbCl_3_ solution was coated onto the UV-LED chip which was already coated with Bi-doped Cs_2_SnCl_6_. The device was then cured at room temperature for 30 min.

##### Measurement and Characterization

Fluorescence emission spectra were conducted on an Ocean Optics spectrometer. Absorbance spectra of samples were measured by using a Shimadzu UV-2550 spectrophotometer. For Bi-doped Cs_2_SnCl_6_ diffuse reflectance (R) spectra were measured by Ocean Optics spectrometer, and the Abs coefficient α was obtained by using the Kubelka–Munk theory (1 − *R*) × (1 − *R*)/2*R*. The excitation spectra and time-resolved PL spectroscopy (TRPL) have been measured by an Edinburgh FLS920 fluorescence spectrometer. The morphology of the QDs was acquired by a FEI Tecnai G2 Spirit TWIN transmission electron microscope (TEM) operating at 100 kV. Scanning electron microscope (SEM) and energy-dispersive X-ray spectroscopy (EDX) measurement have been performed by Quanta 450 FEG. X-ray diffraction (XRD) patterns of perovskites were carried out using a Bruker D8 Advance X-ray diffractometer (Cu Kα: *λ* = 1.5406 Å). The absolute PL QYs of the samples were obtained by a fluorescence spectrometer (FLS920P, Edinburgh Instruments) equipped with an integrating sphere with its inner face coated with BENFLEC. The brightness and efficiency have been measured by ATA-1000 electroluminescence measurement system (Everfine in People’s Republic of China).

## Results and Discussion

**Fig. 1:**
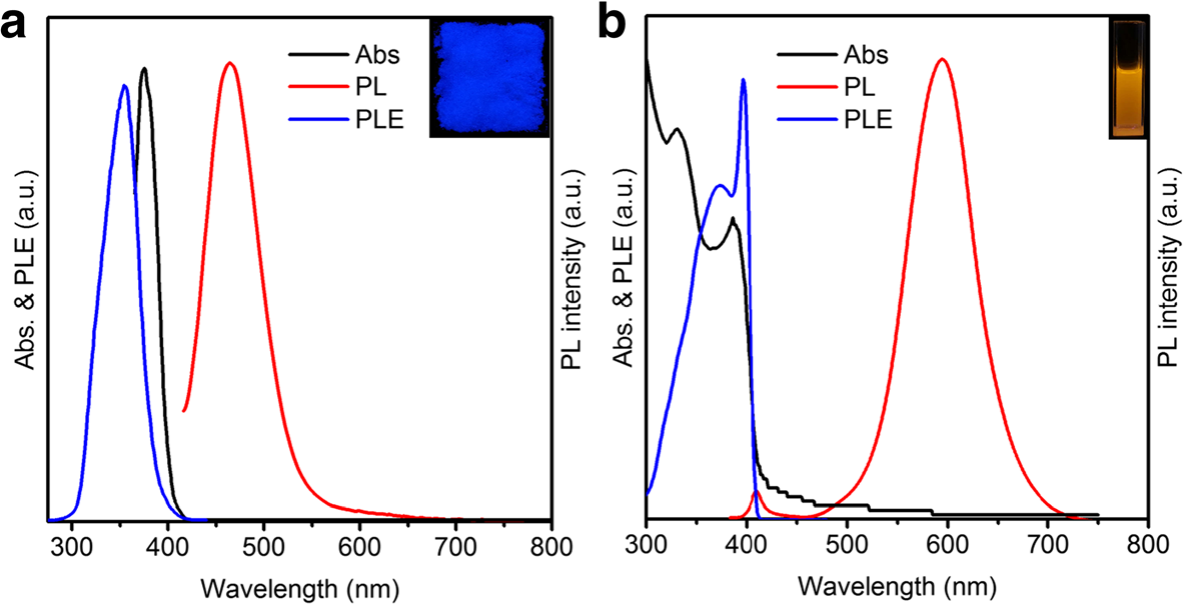
**a** Abs, PL, and PLE spectra of Bi-doped Cs_2_SnCl_6_ QDs. The inset is the photograph of the sample under UV excitation. **b** Abs, PL, and PLE spectra of Mn-doped CsPbCl_3_ QDs. The inset is the photograph of the sample under UV excitation

Bi-doped Cs_2_SnCl_6_ perovskite was synthesized according to the previous approach with little modification [33]. The Abs and PL spectra of Bi-doped Cs_2_SnCl_6_ are presented in Fig. 1a. As shown in Fig. 1a, the sharp Abs peak at around 375 nm could be assigned to the transitions from the defect band (caused by Bi doping) to the host conduction band minimum, which is in good accordance with previous reports [33]. The XRD pattern also indicates the formation of Sn-based perovskite (Fig. 3a). All the diffraction peaks matched well with the Cs_2_SnCl_6_ crystal structure (ICSD #9023), and no impurity phases were detected, which is in good accordance with a previous report [33]. The Bi-doped Cs_2_SnCl_6_ can be excited by UV light (365 nm) and exhibits bright blue light with the PL emission peak located at 465 nm (Fig. 1a). The full width at half maximum (FWHM) of Bi-doped Cs_2_SnCl_6_ is 65 nm, and the QY of Bi-doped Cs_2_SnCl_6_ is up to 76%. The PL excitation (PLE) spectrum of Bi-doped Cs_2_SnCl_6_ has been measured (detected at 465 nm) and shown in Fig. 1a. A broad peak located at 350 nm can be observed in the PLE spectrum of Bi-doped Cs_2_SnCl_6_, which shifts slightly compared to the Abs spectrum. The similar variation was observed by a previous report [33]. In addition, this Bi-doped Cs_2_SnCl_6_ shows excellent stability. After being irradiated for 300 h with UV light, the PL intensity is nearly constant. The perovskite powder can maintain its QY after being exposed to air for 3 months (25 °C, relative humidity 35–50%).

Mn-doped CsPbCl_3_ QDs were prepared according to an established process with minor modification [32]. As shown in Fig. 1b, the Abs peak at around 400 nm is observed, which is assigned to exciton Abs of CsPbCl_3_. Under UV light (365 nm), the QD solution shows a bright orange emission (Fig. 1b, inset). Two peaks are observed in the PL emission spectrum, which center at 405 nm and 595 nm, respectively (Fig. 1b). The peak at 405 nm is assigned to the CsPbCl_3_ host, while the broad emission band with the FWHM at about 80 nm is assigned to Mn^2+^ d-d emission [31, 34]. The QY of our product is reaching to 52%, which is comparable with other reports [32, 35, 36]. The PLE spectrum of Mn-doped CsPbCl_3_ has been measured (detected at 595 nm) and shown in Fig. 1b. The PLE spectrum of Mn-doped CsPbCl_3_ closely follows the Abs spectrum, which demonstrates that the strong PL peak of the Mn emission originates from the exciton of perovskite. The as-prepared QDs show excellent stability, which can preserve their emission properties under ambient atmospheres for at least 3 months (25 °C, relative humidity 35–50%).

**Fig. 2:**
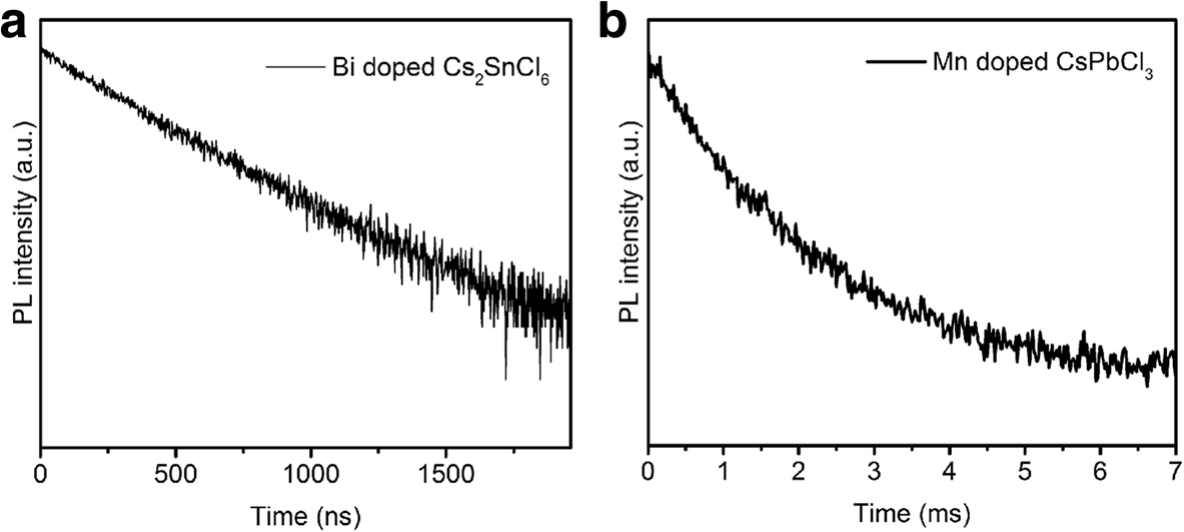
**a** PL decay and fitted curves of Bi-doped Cs_2_SnCl_6_. **b** PL decay and fitted curves of Mn-doped CsPbCl_3_

The PL lifetimes of Bi-doped Cs_2_SnCl_6_ and Mn-doped CsPbCl_3_ were measured using TRPL. As shown in Fig. 2a, the decay curve of Bi-doped Cs_2_SnCl_6_ is fitted well by an exponential function and the lifetime is 375 ns, which is in good accordance with the previous report [33]. As for Mn-doped CsPbCl_3_ QDs, the lifetime is longer (1.7 ms), which supports that it originates from the spin-forbidden ligand field transition of the Mn^2+^ ions [32].

**Fig. 3:**
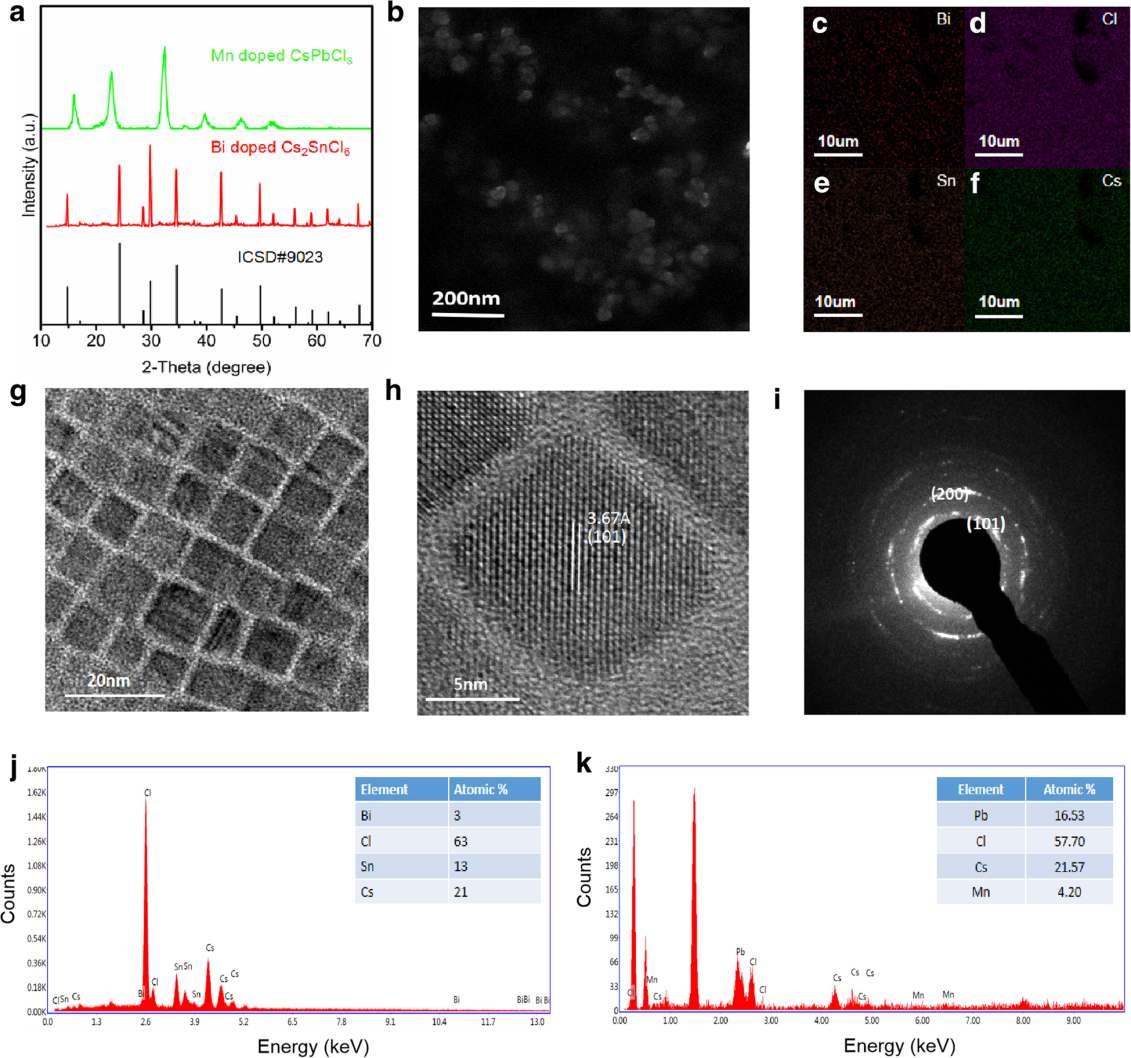
**a** XRD patterns of Bi-doped Cs_2_SnCl_6_ and Mn-doped CsPbCl_3_ QDs, **b** the SEM image of Bi-doped Cs_2_SnCl_6_, **c**–**f** the mapping images of Bi-doped Cs_2_SnCl_6_, **g** TEM image of Mn-doped CsPbCl_3_ perovskite QDs, **h** high-resolution transmission electron microscopy (HRTEM) image of Mn-doped CsPbCl_3_ QDs, **i** elected area electron diffraction (SAED) pattern of Mn-doped CsPbCl_3_, **j** the EDX spectrum and Bi-doped Cs_2_SnCl_6_, and **k** the EDX spectrum of Mn-doped CsPbCl_3_

**Fig. 4:**
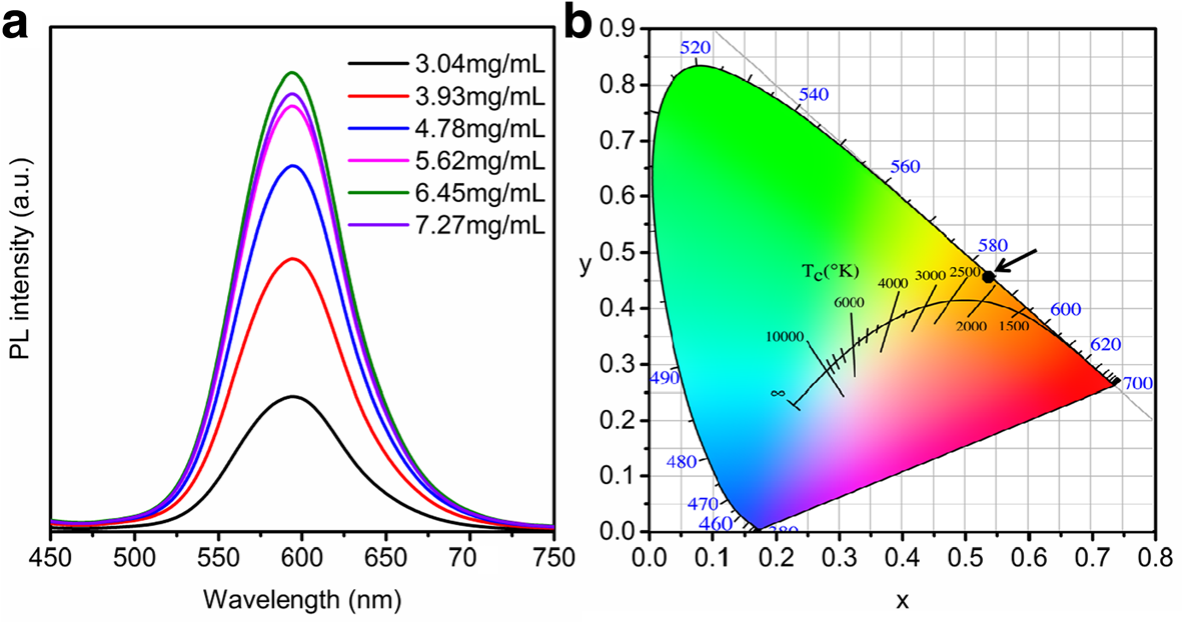
**a** PL spectra of Mn-doped CsPbCl_3_ QDs at different concentration. **b** Color coordinates variation of Mn-doped CsPbCl_3_ QDs at different concentration

Figure 3b shows the SEM image of Bi-doped Cs_2_SnCl_6_ perovskite. The spherical Bi-doped Cs_2_SnCl_6_ perovskite with a diameter of 53 nm can be observed. EDX and mapping images further confirm the presence of Bi in Cs_2_SnCl_6_ (Fig. 3j, c–f). The ratio of Cs, Sn, Bi, and Cl is 1:0.62:0.14:3, which is in good accordance with other reports [33]. Figure 3g shows the TEM image of Mn-doped CsPbCl_3_ QDs. As can be seen, the Mn-doped CsPbCl_3_ QDs show a cubic morphology with an average size of ∼ 12 nm. As can be seen from Fig. 3k, the ratio of Cs, Pb, Mn, and Cl is 1:0.77:0.19:2.68. The HRTEM image displays the lattice fringes of the Mn-doped CsPbCl_3_ QDs, which shows an interplanar distance of 3.67 Å, and matches well with that of the (101) plane (Fig. 3h). The SAED pattern is shown in Fig. 4c. We can see that the QDs possess a tetragonal crystal structure with corresponding (101) and (200) planes (Fig. 3i) [31]. XRD pattern of the Mn-doped CsPbCl_3_ QDs shows that the diffraction peaks are corresponding to the tetragonal phase, which is consistent with the SAED results.

In order to acquire the optimal Mn doping concentration, the emission peak and FWHM variation are analyzed and shown in Fig. 4. It can be seen from Fig. 4a that the PL intensity increases as the concentration of Mn^2+^ is increased from 3.04 to 6.45 mg/mL. Further increasing the Mn^2+^ concentration makes the PL intensity decrease, which is due to the self-Abs effect at high concentration. During the whole process, the PL peak position and the FWHM remains the same. In other words, the change of Mn^2+^ concentration has no effect on the PL emission peak and FWHM, which are also verified by the color coordinates chart (Fig. 4b). No matter how the concentration changes, the color coordinates are basically maintained at (0.535, 0.460) (the black dots). Therefore, the concentration of 6.45 mg/mL is taken as the optimal concentration.

**Fig. 5:**
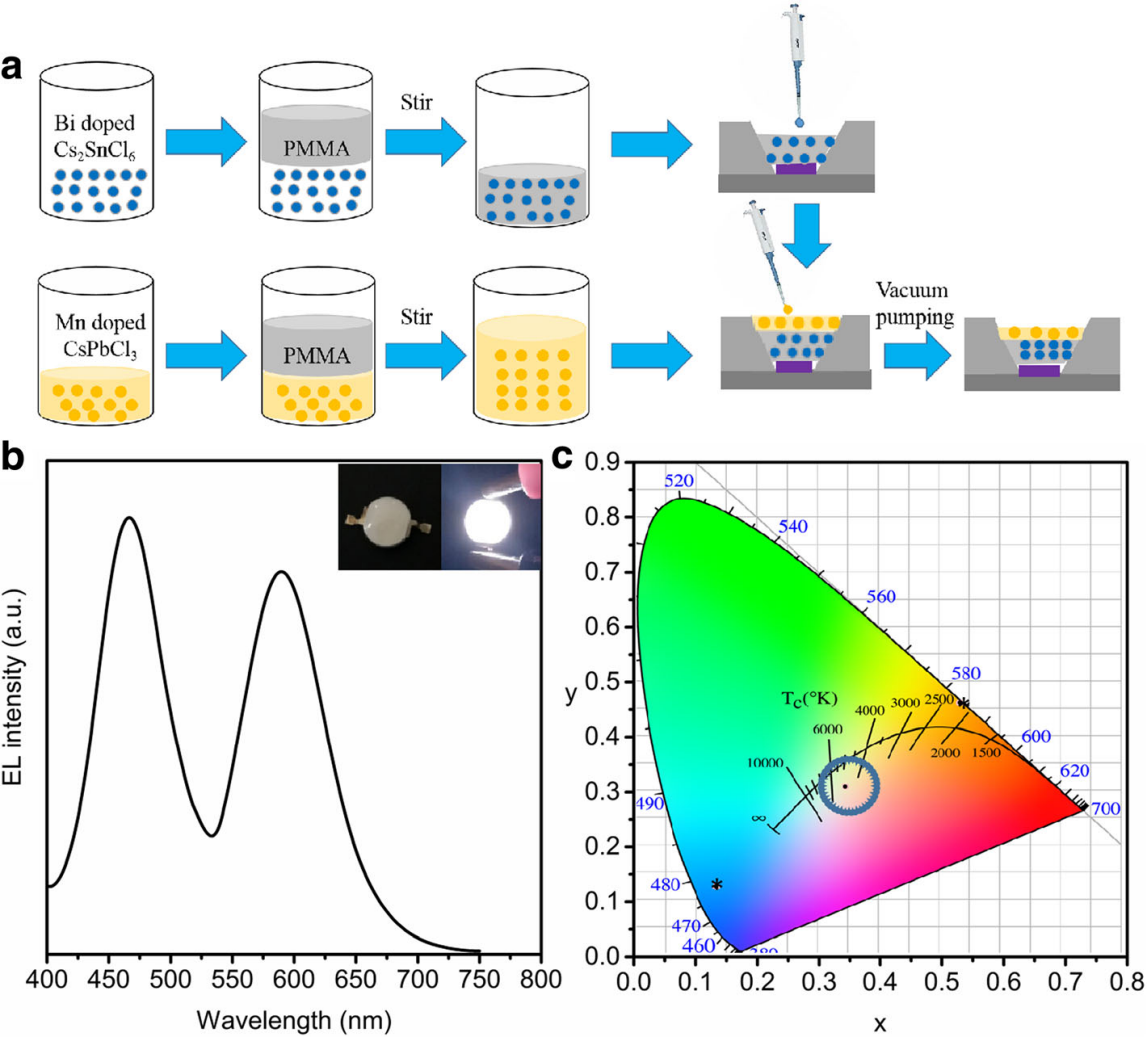
**a** Schematic of the fabrication process of the WLED; **b** electroluminescent (EL) spectrum of WLED; **c** color coordinates of WLED device, Mn-doped CsPbCl_3_ QDs, and Bi-doped Cs_2_SnCl_6。_ (the black dot inside the circle is the white color coordinates and the asterisk represents the blue and orange perovskite). The inserts are the photograph of the WLED

A WLED was fabricated by coating blue-emitting Bi-doped Cs_2_SnCl_6_ powder and orange-emitting Mn-doped CsPbCl_3_ QDs onto a commercially available 365 nm LED chip (Fig. 5a). As shown in Fig. 5b, two obvious peaks can be seen from the EL spectrum of the WLED, which attribute to Bi-doped Cs_2_SnCl_6_ and Mn-doped CsPbCl_3_. The perseverance of these two peaks indicates that no anion exchange and other chemical reactions occur in the fabrication process. In bright white light with color coordinates of (0.334, 0.297), the correlated color temperature of 5311 K can be observed when the WLED is operated at 15 mA (Fig. 5b and c). The highest luminous efficiency and luminance of the WLED reach up to 20.8 lm/W and 78,000 cd m^−2^, respectively, which are comparable with other UV chip-based WLEDs [4, 37–39].

**Fig. 6:**
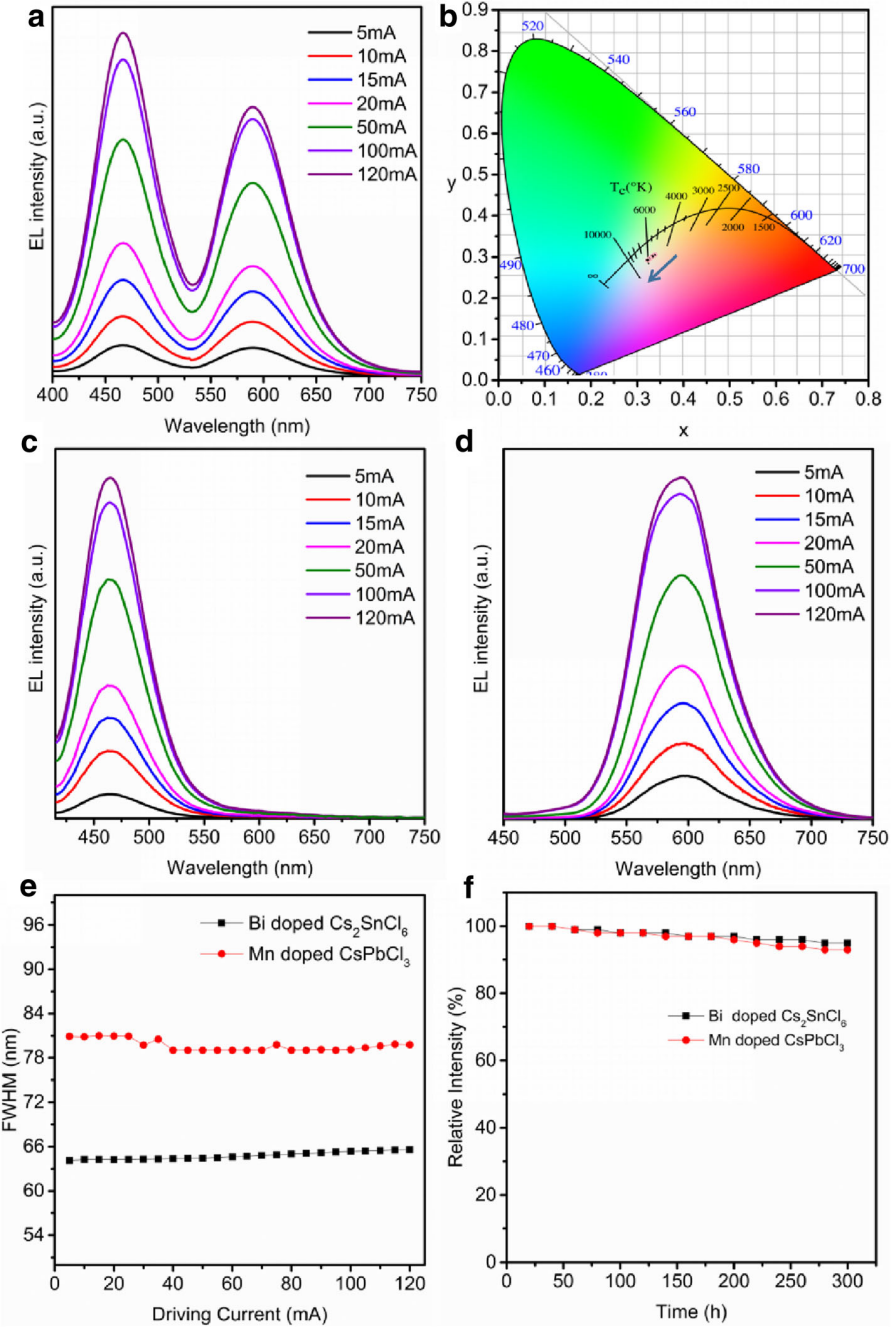
**a** PL spectra of WLED device under different injection currents, **b** chromaticity coordinates change of WLED device under different injection currents, **c** PL spectra of Bi-doped Cs_2_SnCl_6_ LED device under different injection currents, **d** PL spectra of Mn-doped CsPbCl_3_ LED device under different injection currents, **e** the FWHM variation of Bi-doped Cs_2_SnCl_6_ and Mn-doped CsPbCl_3_, **f** the PL intensity variation measured at different working time intervals

The emission spectra of the as-fabricated WLED with the driving currents of 5 mA-120 mA are given in Fig. 6a. The detailed characteristics including color coordinates, CCT, and CRI of the as-fabricated WLED are shown in Table 1. As shown in Fig. 6a, the EL intensities of both peaks increase gradually along with increasing the current and show no saturation. Besides, no obvious shift of the peak position for the EL spectrum occurred under different injection currents. Color coordinates of these PL spectra are shown in Fig. 6b. The chromaticity coordinates show little shift (*x* < 0.02, *y* < 0.02) to the left with increasing the driving currents. We can observe that the EL intensity of Bi-doped Cs_2_SnCl_6_ increases faster than that of the Mn-doped CsPbCl_3_, which can result in chromaticity coordinates moving left. However, the FWHM variation and the shift of emission peak also cause chromaticity coordinates to move. As we discussed above, the emission peak is unchanged with the increase of currents. Because of their broad FWHM, the emission peaks of Bi-doped Cs_2_SnCl_6_ and Mn-doped CsPbCl_3_ overlap, which is difficult to analyze the FWHM variation. Therefore, monochromatic LEDs have been fabricated to analyze each FWHM variation. Figure 6c and d show the emission spectra of Bi-doped Cs_2_SnCl_6_ and Mn-doped CsPbCl_3_ LEDs, respectively. In the wide current range from 5 to 120 mA, no shift of the PL emission peaks occurs, which is in good accordance with the WLED results (Fig. 6c and d). The FWHM variation of the coated LEDs under different currents is shown in Fig. 6e. As can be seen, the FWHMs of Bi-doped Cs_2_SnCl_6_ and Mn-doped CsPbCl_3_ are nearly constant, indicating that the variation of chromaticity coordinates of the WLED only results from the change of intensity of the EL. The difference of EL intensity variation perhaps comes from the different thermal stability of Bi-doped Cs_2_SnCl_6_ and Mn-doped CsPbCl_3_, because the increase of currents could result in the temperature of LED chip increasing. This insignificant change can be further alleviated by adopting a remote-type LED structure. Moreover, the long-term operating stability can be observed from Fig. 6f. After a continuous work of 300 h, the EL intensities of both Bi-doped Cs_2_SnCl_6_ and Mn-doped CsPbCl_3_ are declined less than 10%. Actually, the half-life of the WLED is 3000 h, which is far better than I-containing perovskites [15, 17, 18, 40]. As can be seen from Table 2, after the as-prepared WLED continues working at 15 mA over 50 h, the PL intensity drops to 99% of the original, which is much better than other reports [17, 18, 36, 40–44]. After working 100 hours, the PL intensity only drops to 97%.

**Table 1:** Color coordinates (*x*, *y*), CCT, and *R*_*a*_ of the WLED device under different injection currents

Current (mA)	*x*	*y*	CCT	*R*_*a*_
5	0.340	0.301	5046	82
10	0.339	0.300	5095	81
15	0.334	0.297	5311	80
20	0.329	0.292	5700	79
50	0.328	0.291	5815	78
100	0.327	0.290	5849	78
120	0.324	0.288	6119	77

**Table 2:** Comparison of different WLEDs fabricated by perovskite

Ref.	LED chip	Red component	Green component	Blue component	PL intensity, working time	Current (mA)
1	Blue LED	CsPb (Br/I)_3_	CsPbBr_3_	Blue LED	10 h, 83%	20
2	UV LED	2.7% Ce/9.1% Mn co-doped CsPbCl_1.8_Br_1.2_		UV LED	20 h, 89%	15
3	Blue LED	Mn^2+^-doped CsPb (Cl_0.5_Br_0.5_)_3_	CsPbBr_3_	Blue LED	24 h, 96%	20
4	UV LED	CsPb (Br_0.4_I_0.6_)_3_	CsPb (Br_0.8_I_0.2_)_3_	UV LED	100 h, 73%	15
5	UV LED	CsPbI_3_	CsPb (Br/I)_3_	CsPbBr_3_	30 h, 83.5%	15
6	Blue LED	CSAN:Eu^2+^-EC	CsPbBr_3_-EC	Blue LED	30 min, 80%	20
7	Blue LED	K_2_SiF_6_:Mn^4+^ phosphors	CsPbBr_3_	Blue LED	13 h, 96%	6
8	Blue LED	CaAlSiN_3_:Eu^2+^	CsPb_0.64_Sn_0.36_Br_3_	Blue LED	50 h, 97%	15
This work	UV LED	Mn-doped CsPbCl_3_	–	Bi-doped Cs_2_SnCl_6_	50 h, 99% 100 h, 97%	15

Nowadays, perovskite heterojunctions have been adopted to improve the physical properties of perovskite [45, 46]. Usually, these heterojunctions can integrate the merits of both materials, such as perovskite-polymer bulk heterostructure, perovskite-PbS core-shell structure, and perovskite-plasmonic Au or Ag composite material [47–49], which can enhance efficiency. However, due to the poor stability of perovskite, it is difficult to design and fabricate heterojunction. Besides, these perovskite heterojunctions may be not stable compared to pure perovskite.

## Conclusion

In conclusion, we have combined high-quality blue-emitting bismuth-doped Cs_2_SnCl_6_ perovskite with orange-emitting Mn-doped CsPbCl_3_ QDs to fabricate WLED. Because they all contain the same anion of Cl, anion exchange reaction can be avoided. Besides, orange-emitting Mn-doped CsPbCl_3_ QDs show better stability compared with iodine-containing counterparts. The WLED with color coordinates of (0.334, 0.297) is acquired by tuning the ratio of them. In addition, the WLED show excellent long-term operating stability, which is by far, to our knowledge, the most stable one among perovskite-based WLEDs. We believe our findings will open up new avenues for the exploration of novel lead-free perovskite-based WLED.

## Abbreviations

QDs: Quantum dots
WLEDs: White light-emitting diodes
QY: Quantum yield
PCE: Power conversion efficiency
PL: Photoluminescence
Cs_2_CO_3_: Cesium carbonate
PbCl_2_: Lead (II) chloride
CsCl: Cesium chloride
OA: Oleic acid
ODE: 1-Octadecene
MnCl_2·_(H_2_O)_4_: Manganese chloride tetrahydrate
OAm: Oleylamine
SnCl_2_: Tin chloride
BiCl_3_: Bismuth chloride
PMMA: Polymethyl methacrylate
HCl: Hydrochloric acid
TRPL: Time-resolved PL spectroscopy
TEM: Transmission electron microscope
SEM: Scanning electron microscope
EDX: Energy dispersive X-ray detector
XRD: X-ray diffraction
PLE: PL excitation
FWHM: Full width at half maximum
EL: Electroluminescent
HRTEM: High-resolution transmission electron microscopy
SAED: Selected area electron diffraction
